# Effect of workplace bullying on self-esteem with moderating role of defense styles: a study among pharmacists in Sanaa, Yemen

**DOI:** 10.1080/20523211.2024.2415416

**Published:** 2024-10-18

**Authors:** Anam Mehmood, Umbreen Khizar, Sultan Mehmood Siddiqi, Rabia Mahmood, Mikiyas Amare Getu, Alariqi Reem

**Affiliations:** a School of Biomedical Engineering, Shenzhen University, Shenzhen, Guangdong Province, People’s Republic of China; b Institute of Southern Punjab, Multan, Punjab, Pakistan; c Public Health and Human Nutrition, Tsinghua University, Beijing, People’s Republic of China; d Department of Biochemistry, Bahauddin Zakariya University, Multan, Punjab, Pakistan; e Woldia University, Weldiya, Ethiopia; f Microbiology Department, Al-Razi University, Sana’a, Yemen; g Microbiology Department, Amran University, Amran, Yemen

**Keywords:** Workplace bullying, self-esteem, defense styles, pharmacists

## Abstract

**Background:**

Workplace bullying is a widespread occurrence that has been identified as a significant concern in enterprises all around the world. The research aimed to examine the impact of workplace bullying on self-esteem among pharmacists with the moderating role of defense styles.

**Method:**

This study utilised stratified random sampling to include 498 pharmacists from public and private hospitals in Sanaa, Yemen. Data was collected through structured questionnaires on demographics, workplace bullying (NAQ-R), self-esteem (Rosenberg scale), and defense styles (DSQ-40). Data analysis involved descriptive statistics, regression, correlation, T-tests, and moderation analysis.

**Results:**

Results of the current study showed that workplace bullying has a significant negative impact on self-esteem, and defense styles do not moderate self-esteem and workplace bullying. Female pharmacists have higher workplace bullying and lower self-esteem. Gender differences in defense styles have insignificant results.

**Conclusion:**

This research supports overcoming workplace bullying among employees in any organisation. Comprehensive anti-bullying policies must be developed and implemented to create a safe and supportive workplace for pharmacists. Anti-bullying strategies that promote gender equality address power disparities and guarantee that female employees have access to particular resources for support should incorporate gender-sensitive practices. In order to reduce the adverse effects of workplace bullying on mental health and boost the self-esteem of victims by offering counseling services, frequent training sessions, and seminars. It is also helpful in enhancing self-esteem and improving interpersonal relationships among employees.

## 1. Background

### 1.1. Workplace bullying

Workplace bullying is a widespread occurrence that has been identified as a significant concern in enterprises all around the world. Acts that cause irritation, offense, social exclusion, or adversely affect an individual's capacity to perform their duties are classified as workplace bullying (Farley et al., 2023a). It is a type of mistreatment that occurs over an extended period and can take the form of social isolation, physical or verbal abuse, or psychological harassment (Azmat et al., 2023). Workplace bullying is a significant societal matter that has been the focus of several research studies (Aquino & Thau, 2009; Gupta et al., 2020; Nielsen & Einarsen, 2018). An elevated risk of hypertension and mental health issues is associated with ongoing exposure to stressful situations brought on by workplace bullying (Jones, 2017). Employee's well-being is negatively impacted by workplace bullying, which sends them into a state of mind when bosses exhibit negative behaviours, causing them to feel dread, worry, and anxiety (Farley et al., 2023b). This empowering work setting exacerbates bullying, a lack of collaboration, the perception that the workplace is unsafe, and bullying cultures, which are ones in which the behaviour is acknowledged to exist and seems to be supported by people in positions of authority (Wilson, 2016). Prior research found the detrimental influence of bullying in the workplace on job engagement mediated by the denial of core needs and the resulting lack of intrinsic motives to work (Goodboy et al., 2020). Bullying victims can feel ashamed, guilty, and worthless, and in extreme situations, self-hatred, which demotivates them and lowers their self-respect and self-esteem. Due to this, the employee may lose interest in their job or become socially isolated. As a result, they may be unable to strike a work-life balance and may consider abandoning their job (Villa et al., 2020).

### 1.2. Workplace bullying and self-esteem

Self-esteem is essential to human cognition since people constantly strive to nurture it, impacting how they act and feel (Arand, 2019). High self-esteem is related to high resilience to Stress and better coping strategies, whereas low self-esteem may aggravate the effects of negative experiences like bullying (Li et al., 2020). Studies stated that workplace bullying can significantly alter self-esteem and self-efficacy, ultimately causing psychological issues, including anxiety and depression (Noor et al., 2016; O’Moore & Kirkham, 2001; Van den Brande et al., 2021). When employees face adversity, such as bullying at work, they are likelier to use their most outstanding personality features to counteract adverse workplace behaviours (Chen et al., 2020).

### 1.3. Role of defense styles

Defenses are mechanisms that moderate the attachment style to decrease the feelings of distress connected with negative anticipations, both at an interpersonal and interpersonal level. Defense style has been inspected as an individual's static changes focused on stressful experiences, precisely tension, developing from a psychological imbalance linked to the insight of internal and external stressors (Kobak & Bosmans, 2019). Different defense styles were significantly connected with the observance of general principles. Specifically, adopting mature defenses instead of neurotic or immature defenses can have a profound impact on mental health. Mature defense systems aid in crisis resilience, significantly altering mental health (Marčinko et al., 2020). In the paradigm of workplace bullying, defense mechanisms act as coping strategies, helping individuals to navigate hostile environments. However, extravagant dependence on these mechanisms can also indicate dysfunctional coping and poorer mental health outcomes (Petraglia et al., 2018). Researchers view workplace bullying as a job demand requiring high cognitive and emotional effort, negatively impacting well-being (Hoprekstad et al., 2019; Ng et al., 2022). A Spanish study conducted by Elena Losa Iglesias et al. (2012) reported that Spanish nurses who suffered from bullying behaviors had lesser levels of self-esteem. Li et al. (2020) found that self-esteem is a crucial personality variable. People with low self-esteem possess poor coping abilities and sensitive personalities, which cause them to become targets of workplace bullying. Bakken et al. (2021) and Barnett et al. (2022) explored in two different studies that pharmacists who faced bullying have shown decreased work satisfaction (Bakken et al., 2021; Barnett et al., 2022). However, these researches have primarily focused on bullying in the pharmacy profession. The most common impacts of workplace bullying are linked to increasing stress levels, adverse effects on emotional health, and job dissatisfaction. Female participants more frequently reported workplace bullying compared to male participants. Klein and coworkers found that People from the white race were less likely to report workplace bullying compared to other races(Klein et al., 2021).

Previous research has highlighted the prevalence of workplace bullying across various professions, but there needs to be more studies focusing specifically on pharmacists. In the medical field, pharmacists are essential. Workplace challenges they face include meeting deadlines, dealing with demanding customers, and ensuring patient safety. The presence of workplace bullying with other challenges might harm pharmacists’ job satisfaction levels by elevating the risk of job burnout. In the healthcare industry, it is imperative to retain skilled staff. Research on the effect of workplace bullying on pharmacists’ self-esteem is crucial since low self-esteem can undermine one's sense of professional worth and empowerment. According to social identity theory, bullying can damage a person's social identity and self-esteem, particularly in work-related settings. Defense mechanisms are viewed as coping mechanisms that mediate stress reactions, according to Coping Theory by Lazarus and Folkman. By processing bullying differently, pharmacists with defense mechanisms might lessen its detrimental effects on self-esteem. Given their pivotal role in healthcare, it is essential to investigate how workplace bullying affects this group. The current research aims to explore the correlation between workplace bullying, self-esteem, and defense styles among pharmacists in private and public hospitals in Sanaa, Yemen. The study focuses on pharmacists in Sanaa, Yemen, due to the region's healthcare challenges, including insufficient facilities and conflict, leading to public health emergencies. Pharmacists are crucial in managing resources, providing primary healthcare, and educating the public. Their strategic position and essential services make them ideal subjects for studying successful healthcare delivery in crisis-affected countries. The findings from this research provide a fundamental understanding of the underlying forces of workplace bullying and its effect on self-esteem and coping mechanisms, contributing to a better understanding of mitigating its adverse effects. The primary objectives of this study are to find the prevalence of workplace bullying among pharmacists in private and public hospitals in Sanaa, Yemen, to find out the effect of workplace bullying on self-esteem among these pharmacists, and to check the role of defense mechanisms in moderating the relationship between workplace bullying and self-esteem. The present study has the following questions to address:

1. Is there a significant prevalence of workplace bullying among pharmacists in Sanaa, Yemen?
2. Is workplace bullying negatively associated with self-esteem among pharmacists?
3. Do defense mechanisms moderate the relationship between workplace bullying and self-esteem?

This significant study fills a gap in the existing research by focusing on pharmacists, a critical but under-explored group in the context of workplace bullying. By identifying the occurrence and consequence of bullying on self-esteem and investigating the role of defense mechanisms, the study offers insightful information that can guide the creation of interventions targeted at mitigating the adverse effects of workplace bullying. Such interventions are crucial for fostering a healthier work environment, enhancing job satisfaction, and improving patient care outcomes.

## 2. Method

### 2.1. Sample selection

A stratified random sampling style targeted the pharmacists of private and public hospitals in Sanna, Yemen. Each hospital was a stratum. The stratification was supposed to get a representative sample from various hospitals. Four hundred ninety-eight participants (male 269, female 239) participated in this study. In order to get the optimal sample size for current research, we conducted a power analysis with a G*power formula, aiming for 80% power with a significance level of 0.05. This analysis was grounded on an expected moderate effect size (Cohen’s *d* = 0.5). The individuals who met the requirements to be included in this research were in the age range of 25 and 55, registered hospital paramedical staff without psychiatry history, and were willing to participate. Exclusion criteria were those on long-term leave or retired and unwilling to participate. An online survey was conducted to minimise the chances of bias and to ensure confidentiality. Written Informed consent was obtained before participation, and participants were aware of our research. The Ethics Review Committee of Sanaa University, Yemen, approved the study.

### 2.2. Procedure

The period for data collection was from January 2024 to July 2024. The data was collected through a structured questionnaire that involved demographic information and psychological measures. The questionnaire was sent online, including professional email addresses, hospital intranet systems, and in person. This approach was employed to facilitate broad accessibility for paramedical staff. All records were kept in a locked computer and under strict supervision.

### 2.3. Psychological measures

A structured questionnaire package designed to obtain demographic information (age, gender, marital status, level of education, year of experience, income level, place of stay, type of hospital, employment status, and shift work) along with Negative Acts Questionnaire-Revised, Rosenberg self-esteem scale and Defense style questionnaire were sent to the participants. The participants were requested to complete the survey. Every questionnaire was designed to be self-reporting.

**Negative Acts Questionnaire-Revised (NAQ-R):** The Negative Acts Questionnaire-Revised (NAQ-R) (Nam et al., 2010) accessed workplace bullying. It consists of 22 items. Three underlying factors are measured: personal, work-related, and physically intimidating forms of bullying. The responses to all items are averaged. Greater scores mean more significant workplace bullying. In the present research, the scale indicated good reliability (Cronbach’s *α* = 0.96).

**Rosenberg's Self-Esteem Scale:** Self-esteem was measured using Rosenberg's self-esteem scale (Rosenberg, 1965). This ten-item measure evaluates one's overall sense of self-worth by gauging positive and negative thoughts about oneself. The participants used a 4-point rating scale, from strongly disagree to agree, to rate each item strongly. Better scores denoted a better degree of self-esteem. The measure demonstrated good reliability in our investigation. (Cronbach’s *α* = 0.86).

**Defense Style Questionnaire (DSQ-40):** The Defense Style Questionnaire (DSQ-40) was used to measure defense style, which is a self-report inventory (Petraglia et al., 2009; Saint Martin et al., 2013). It measures specific defense mechanisms. The defense-style questionnaire was initially constructed in 1980 by Bond, Gardner, Christian, and Sigal (Bond et al., 1983). This questionnaire includes various statements about personal attitudes, with a rating on a rating scale. Using the nine-point scale, circle one of the numbers on the scale beside each argument to indicate the level of agreement or disagreement with it. A score of 5 indicates neither agree nor disagree with the sentence, while a score of 3 indicates disagree moderately. A total of 9 demonstrate strongly agree. A good reliability was indicated by this questionaire in our study (Cronbach’s *α* is 0.89).

### 2.4. Statistical analysis

The collected data was organised, scored, analysed, and interpreted. Demographic data, including age, gender, marital status, educational level, year of experience, income level, place of stay, type of hospital, employment status, and shift work, were recorded as numbers and percentages. The missing data was less than 2% and was controlled through mean attribution. Descriptive statistics, Regression, correlation, T-tests, and moderation analysis were conducted. SPSS (26.0 v) was used to perform the analysis with a significance level of *p* < 0.05.

## 3. Results

### 3.1. Demographics data of participants

The present study comprised 498 participants from different hospitals in Sanna, Yemen. Most participants were 36–45 years old (59.44%), with a considerable proportion of males (52.01%). Regarding marital status, most were married (61.24%), and 20.89% were separated. The distribution displays a variety of educational backgrounds, with most individuals possessing a Pharm.D. degree (44.98%). The years of experience and income levels varied among the participants. As for their place of stay, 12.05% of the participants lived in hostels, 38.15% in private rooms, and 33.53% in their own houses. The type of institution where they were employed showed that 63.65% worked in public institutions.

Regarding employment status, most of the participants were full-time employees (65.26%). Regarding shift work, most have proper night and day shifts, and few have rotational shifts. These detailed demographic insights provide valuable information about the characteristics of the study participants (Table 1).

**Table 1.** Demographic characteristics.

Demographic characteristics	Number of participants (%)
**Age**	
25–35	92 (18.47%)
36–45	296 (59.44%)
46–55	110 (22.09%)
**Gender**	
Male	259 (52.01%)
Female	239 (47.99%)
**Marital status**	
Single	89 (17.86%)
Married	305 (61.24%)
separated	104 (20.89%)
**Educational level**	
B. Pharm.	108 (21.69%)
Pharm.D.	224 (44.98%)
Masters in Pharmacy	125 (25.10%)
Diploma	41 (8.23%)
**Year of experience**	
0–5 years	124 (24.90%)
6–10 years	189 (37.95%)
11–20 years	117 (23.49%)
21 + years	68 (13.65%)
**Income level (Yemeni Rial)**	
<30,000 (Low)	102 (20.48%)
40,000–90,000 (Middle)	221 (44.38%)
>100,000 (High)	175 (35.14%)
**Place of stay**	
Hostel	60 (12.05%)
Private Room	190 (38.15%)
Own house	167 (33.53%)
**Type of Hospital**	
Public	317 (63.65%)
Private	181 (36.35%)
**Employment status**	
Full-time	325 (65.26%)
Part-time	173 (34.74%)
**Shift work**	
Night	259 (52.01%)
Day	199 (39.96%)
rotational	40 (8.03%)

### 3.2. Strength of the correlation among workplace bullying, self-esteem, and defense styles

The relationship between workplace bullying, self-esteem, and defense styles was analyzed using correlation (Table 2). Workplace Bullying has a noteworthy negative relationship with Self-esteem (*r *= −.24, *p *< 0.01) and has a significant positive correlation with Defense Style (*r* = 0.42, *p *< 0.01). Self-esteem correlates negatively with Defense style (*r *= −.23, *p *< .05).

**Table 2.** Descriptive statistics and correlations matrix for study variables.

Variables	Mean	Standard Deviation	1	2	3
1. Workplace Bullying	42.70	15.98	–		
2. Self-esteem	23.72	2.51	−.24**	–	
3. Defense Style	217.17	32.65	.42**	−.23*	–

*p < .05. **p < .01.

### 3.3. Impact of workplace bullying on self-esteem

The impact of workplace bullying on self-esteem among pharmacists was analysed through Regression (Table 3). The *R^2^* value of .09 indicates that 09% of the variance is in the outcome variable with *F* (1, 498) = 7.13, *p* < .001. The findings revealed that workplace bullying negatively predicted self-esteem (*β* = −.24, *p* < .001).

**Table 3.** Regression coefficients of workplace bullying on self-esteem.

Variables	*B*	*β*	SE
Constant	24.39**		0.66
Workplace bullying	−0.024**	−0.24	0.01
*R^2^*	0.09		

**p < .001.

### 3.4. Comparison of workplace bullying, self-esteem, and defense styles between male and female

A t-test was used to compare female and male participants based on workplace bullying, self-esteem, and defense styles (Table 4). Results revealed significant mean differences in Workplace Bullying with *p *< .001. Findings showed that females showed higher scores on Workplace Bullying (*M* = 46.21, *SD* = 15.75) compared to males (*M* = 39.00, *SD* = 15.44). Mean differences in Self-esteem were significant with *p *< .001. The finding showed that males had higher self-esteem scores (*M* = 23.75, *SD* = 2.18) than females (*M* = 23.70, *SD* = 2.81). While non-significant mean differences in Defense styles with *p *< .05 were observed.

**Table 4.** t-test for comparison of workplace bullying, self-esteem, defense styles between male and female.

Variables	Male		Female		*p*	Cohen’s *d*
	*M*	*SD*	*M*	*SD*		
Workplace Bullying	39.00	15.44	46.21	15.75	.007	.49
Self Esteem	23.75	2.18	23.70	2.81	.008	.52
Defense Styles	220.58	31.51	213.65	33.68	.235	.22

### 3.5. Defense style’s role as moderation between workplace bullying and self-esteem

Table 5 shows the moderation of defense styles concerning workplace bullying and self-esteem. In Model 1, the *R^2^* value .09 revealed that the predictors explained a 09% variance in the outcome. Workplace bullying negatively predicts self-esteem (*β* = −.19, *p* < .01). Defense styles also show a negative effect on self-esteem (*β* = −.157), although this effect is not statistically significant. In Model 2, the *R^2^* value .092 revealed that the predictors explained a 09% variance in the outcome with *p *< .001. The finding revealed that workplace bullying (*β* = .18, *p *< .01), defense styles (*β* = .16), and workplace bullying x defense styles negatively predicted self-esteem (*β* = .007). The Δ*R^2^* value of .001 revealed a 0.1% change in the variance of model 1 and model 2. Findings showed that defense style did not moderate the relationship between workplace bullying and self-esteem.

**Table 5.** Moderation of defense styles between workplace bullying and self-esteem.

Variables	Model 1			Model 2		
	*B*	*β*	*SE*	*B*	*β*	*SE*
Constant	22.76***		.22	22.77***		.24
Workplace Bullying	−.46**	−.19**	.24	−.45**	−.18**	.25
Defense Styles	−.38	−.157	.24	−.38	−.16	.25
Workplace Bullying × Defense Styles				−.02	−.007	.20
*R^2^*		.09			.092	
Δ*R^2^*					.001	

***p < .001. **p < .01.

## 4. Discussion

The primary objective of the current research was to examine the connection between workplace bullying, self-esteem, and defense styles among pharmacists in private and public hospitals in Sanaa, Yemen. The findings provide important insights into these dynamics, revealing significant correlations and the impact of workplace bullying on self-esteem.

The effect of workplace bullying on self-esteem was explored, suggesting that bullying is linked with low levels of self-esteem among pharmacists. Research conducted on Spanish physicians found that workplace bullying has detrimental effects on self-esteem and self-worth in the workplace (Iglesias & de Bengoa Vallejo, 2014). The relationship between bullying at workplace self-esteem and defense styles was examined. The significant positive correlation between workplace bullying and defense styles proposes that nurses experiencing higher levels of bullying are inclined to use defense mechanisms frequently, potentially as a coping strategy (Arand, 2019). Furthermore, the inverse relationship between self-esteem and defense styles bespeaks that higher self-esteem is related to fewer defense mechanisms, suggesting that individuals with higher self-esteem may cope with Stress more effectively without resorting to defensive behaviours. Poor self-esteem is connected with an increased likelihood of workplace bullying (Li et al., 2020). In regression results, we explored that workplace bullying significantly predicts lower self-esteem. This finding underscores the detrimental outcome of workplace bullying on self-esteem. Previous studies reported that bullying in the workplace can lead to decreased self-esteem, depression, anxiety, and other adverse mental outcomes (Nielsen & Einarsen, 2018). The counter impact on self-esteem is probably due to the demeaning and sabotaging nature of bullying.

Regarding Gender differences, female participants reported higher levels of workplace bullying, and male participants reported slightly higher self-esteem. It proposed that female pharmacists may be much more vulnerable to workplace bullying, which could be attributed to gender differences and power imbalances in the workplace (Salin, 2003). We found no significant dissimilarities in using defense styles between males and females, which aligns with the previous study (Petraglia et al., 2015). The study looks into gender differences in bullying experiences and self-esteem, with a focus on the role of sociocultural influences. It demonstrates how societal norms and expectations regarding gender can prevent males from reporting bullying, whereas societal pressure on females to comply with specified habits can exacerbate the psychological impact of bullying. This study attempts to find complicated mechanisms that influence gender-specific bullying experiences and provide better therapeutic choices.

Defense styles as a moderator between workplace bullying and self-esteem were assessed. No prior research backs up the moderation of defense style in the relationship between workplace bullying and self-esteem. While workplace bullying and defense styles negatively predicted self-esteem, the fundamental interaction was insignificant. It highlights that although defense mechanisms are adopted in response to bullying, they do not fend off the negative impact of bullying on self-esteem. The intensity of bullying may be one factor contributing to this non-significant moderation. Bullying may have a powerful effect that defending strategies cannot mitigate it. Because of the difficulties they face in the profession, pharmacists may manage anxiety situations differently, which can lessen the moderating benefits of defense methods. The sample may be homogeneous in age, professional experience, or cultural background, which could lead to a lack of variation in defense styles and an inability to detect moderating effects.

Our outcomes highlight the critical need for effective workplace policies and interventions to forbid and address bullying, mainly focusing on supporting female employees who may be more adversely affected. Hospital management must create an anti-bullying policy and routinely inform all staff members about it to foster a productive work environment. Regular evaluations of the working environment and surveys can be used to track how well anti-bullying policies are working. In a nurturing setting, pharmacists are more likely to talk candidly about bullying and take swift action when necessary. A conflict resolution team can settle disputes before they become bullying. Acknowledging positive behaviour can help pharmacists in their career advancement. Moreover, fostering a supportive work environment can help mitigate the adverse effects of bullying and enhance employee self-esteem.

### 4.1. Practical implications

This research has numerous practical suggestions for hospital management and policymakers. Development and implementation of comprehensive anti-bullying policies are pivotal to creating a supportive and safe work environment. Implementation of these anti-bullying policies is essential. Every employee should be given a clear explanation of what constitutes verbal, physical, and emotional abuse and how to report it. Practical examples, dispute resolution techniques, and unambiguous guidelines for incident reporting should all be included in this training session for pharmacists. Bullying should be treated with zero tolerance at all levels of the health care system. Platforms that allow for anonymity should be offered for privacy reasons.

Providing counseling services, regular training sessions, and seminars for victims of workplace bullying can lessen the adverse effects on mental health and improve self-esteem. Training and awareness campaigns are also essential. Gender-sensitive approaches should be integrated into anti-bullying strategies that will lead to promoting gender equality, dealing with power imbalances, and ensuring that female workers have an approach to specific support resources.

### 4.2. Limitations and future research

This research offers insightful information, but it is essential to understand its limitations. The capacity to deduce causation is restricted by the cross-sectional design. Longitudinal studies are required to investigate the temporal correlations between workplace bullying, self-esteem, and defense styles. The study also used self-report measures, which are vulnerable to biases in response. The forthcoming study could incorporate multiple data sources, such as peer reports or objective measures of workplace bullying. Other sociocultural factors such as education, age, professional experience, and social class can also be examined as moderators. Exploring the effectiveness of various interventions in controlling bullying and its negative effects in different cultural and organisational contexts can provide worthy information for formulating effective strategies. Further research should examine whether the impact of workplace bullying can moderated by other psychological factors, such as resilience and coping methods.

### 4.3. Conclusion

In conclusion, the present research highlights the noteworthy negative effect of workplace bullying on self-esteem among pharmacists in Sanaa, Yemen. This study offers insight into how a hostile work environment can impact one's self-concept, which makes it a valuable theoretical addition. This research advances the ideas that describe how particular dynamics within the healthcare system might interact with bullying and affect self-esteem. These findings emphasised the need for effective interventions to address bullying and support victims. By fostering a satisfactory and inclusive work environment, hospitals can enhance their staff's mental health and professional well-being, leading to better patient care and organisational performance. Addressing bullying in the workplace is not only a matter of individual well-being but also an organisational task to handle it. Bullying can cause less job satisfaction, increased absenteeism, and excellent employee turnover rates that can adversely affect hospital operations and patient care. Therefore, implementing comprehensive strategies to address and prevent workplace bullying is essential for maintaining over-productive, healthy, and supportive work environments. Encouraging a workplace free from bullying is not only morally right; it is also strategically necessary for the long-term expansion and sustainability of the organisation.
