# Correction

**DOI:** 10.1080/20523211.2026.2636423

**Published:** 2026-03-16

**Article title:** Effect of workplace bullying on self-esteem with moderating role of defense styles: a study among pharmacists in Sanaa, Yemen

**Authors:** Anam Mehmood, Umbreen Khizar, Sultan Mehmood Siddiqi, Rabia Mahmood, Mikiyas Amare Getu and Alariqi Reem

**Journal:** *Journal of Pharmaceutical Policy and Practice*

**Bibliometrics:** Volume 17, Number 1, 2415416

**DOI:** https://doi.org/10.1080/20523211.2024.2415416

The authors requested several corrections throughout the article. These revisions primarily involve slight adjustment to the phrasing, ensuring that discussion is framed around the mediation of defense mechanisms. The authors believe that these changes will improve the article's precision and clarity.

These changes include:

- Updating the article title to: Effect of workplace bullying on self-esteem with mediating role of defense mechanism: a study among pharmacists
- Changing all uses of the words ‘moderating’ and ‘moderate’ to ‘mediating’ and ‘mediate’ throughout.
- Changing all uses of the phrase ‘defense style to ‘defense mechanism’ throughout.
- Table 5 was replaced with an updated Table.

